# Prevalence of beliefs about actual and mythical causes of cancer and their association with socio-demographic and health-related characteristics: Findings from a cross-sectional survey in England

**DOI:** 10.1016/j.ejca.2018.03.029

**Published:** 2018-11

**Authors:** Lion Shahab, Jennifer A. McGowan, Jo Waller, Samuel G. Smith

**Affiliations:** a Department of Behavioural Science and Health, University College London, London, WC1E 6BT, UK; b Institute of Child Health, University College London, London, WC1E 6BT, UK; c Leeds Institute of Health Sciences, University of Leeds, Leeds, LS2 9NL, UK

**Keywords:** Cancer, Cancer beliefs, Cancer awareness, General population, Cancer myths

## Abstract

**Background:**

Literature on population awareness about actual causes of cancer is growing but comparatively little is known about the prevalence of people's belief concerning mythical causes of cancer. This study aimed to estimate the prevalence of these beliefs and their association with socio-demographic characteristics and health behaviours.

**Methods:**

A survey containing validated measures of beliefs about actual and mythical cancer causes and health behaviours (smoking, alcohol consumption, physical activity, fruit and vegetable consumption, overweight) was administered to a representative English population sample (N = 1330).

**Results:**

Awareness of actual causes of cancer (52% accurately identified; 95% confidence interval [CI] 51–54) was greater than awareness of mythical cancer causes (36% accurately identified; 95% CI 34–37; *P* < 0.01). The most commonly endorsed mythical cancer causes were exposure to stress (43%; 95% CI 40–45), food additives (42%; 95% CI 39–44) and non-ionizing electromagnetic frequencies (35%; 95% CI 33–38). In adjusted analysis, greater awareness of actual and mythical cancer causes was independently associated with younger age, higher social grade, being white and having post-16 qualifications. Awareness of actual but not mythical cancer causes was associated with not smoking and eating sufficient fruit and vegetables.

**Conclusions:**

Awareness of actual and mythical cancer causes is poor in the general population. Only knowledge of established risk factors is associated with adherence to behavioural recommendations for reducing cancer risk.

## Highlights

• Approximately half of known lifestyle-related risk factors were not recognised by the general public in England.
• Belief in mythical risk factors was common, particularly stress, food additives and electromagnetic frequencies.
• Knowledge of actual but not mythical cancers causes was associated with health-protective behaviours.

## 1 Introduction

Approximately one third to one half of cancer diagnoses are preventable by changes to lifestyle behaviours, amounting to at least 1.1 million avoidable cancer cases per year in Europe [1], [2]. As outlined in the latest (4th) European Code Against Cancer (ECAC), established cancer risk factors include active and passive smoking [3], alcohol consumption [4], overweight and obesity [5], physical inactivity [6], poor diet [7] and exposure to ultraviolet radiation [8] and human papillomavirus (HPV) [9]. Part of the 12 ECAC lifestyle recommendations to reduce cancer risk, therefore, features advice to not smoke and have a smoke-free home, to maintain a healthy body weight, active lifestyle and healthy diet, to avoid too much sun (especially for children), to limit alcohol consumption and to take part in HPV vaccination programmes (for girls) [10]. Yet, in Europe, more than one third of adults fail to meet aerobic activity guidelines [11], more than a quarter continue to smoke [12] and more than half are overweight [13]. In the United Kingdom (UK), three quarters of the population do not eat the recommended amount of fruit and vegetables [14], and 31% of men and 16% of women drink alcohol above recommended levels [15]. Accurate public awareness of cancer risk factors is an important component of informed decision-making about lifestyle behaviour change.

Data from multiple European countries indicate poor awareness of the link between lifestyle factors and cancer risk [16], [17], [18], [19], [20], [21], [22], [23]. Public understanding of cancer risk factors is likely to include beliefs in mythical risk factors with no known association with cancer development. Baseline findings from a nationwide UK awareness campaign reported that approximately one third of the sample endorsed stress as a cause of cancer, and more than a quarter agreed that living near power lines increased cancer risk [17]. Similar beliefs have been reported among Dutch bladder cancer survivors, although the prevalence was markedly lower [24]. Endorsement of incorrect risk factors is particularly high among underserved populations, including lower socio-economic status groups, people with lower levels of education and ethnic minorities [16], [17], [20], [23], [25].

The way in which we think about potential risk factors for disease can trigger risk reduction behaviours [26]. For example, causal beliefs about cancer can influence the use of complementary therapies, diet and lifestyle and treatment decision-making [27], [28], [29]. However, if behavioural efforts are misdirected towards reducing risk from mythical cancer causes, behaviour change for known cancer causes may be less likely to occur. For instance, melanoma patients report factors unrelated to sunburn such as stress as a cause of their cancer [30]. Moreover, the tobacco industry in the past deliberately funded work on spurious risk factors to detract from actual causes of neoplastic and cardiovascular diseases, namely smoking [31].

The Cancer Awareness Measure (CAM) is a validated tool of known cancer risk factors [32], but no measure has been developed to accurately identify the range of beliefs people hold about mythical cancer causes. To address this, we developed the CAM—MYthical Causes Scale (CAM-MYCS) [33]. The aim of the current analysis is to use nationally representative data to report the prevalence of beliefs about mythical cancer causes and the socio-demographic correlates of holding these beliefs. We also examined the associations of CAM and CAM-MYCS with cancer-related health behaviours. These data will help to characterise the population and will inform future cancer prevention research and practice.

## 2 Materials and methods

### 2.1 Study design

Data come from the Attitudes and Beliefs about Cancer-UK Survey (ABACUS), a large population-based cross-sectional omnibus survey in England carried out by TNS Research International between January 2016 and March 2016. This survey creates sample points using the 2001 Census small-area statistics and the Postcode Address File (stratified by social grade and Government Office Region) for random location sampling. Quotas for age, gender, children in the home and working status are set for each location, and three doors are left between each successful interview. Data were collected using computer-assisted face-to-face interviews by a trained interviewer in the respondents' homes. The study was approved by the University College London Ethics Committee (Project ID 5771/002), and participants consented to participate at the start of the omnibus survey.

### 2.2 Participants

A total of 1990 adults took part in the ABACUS survey of whom all completed the CAM but only a randomly selected subsample of 1348 adults was also asked to complete the CAM-MYCS to determine whether this new measure influenced responses to the original CAM [33]. Participants who did not respond to all cancer belief items were excluded from analysis, resulting in a final sample size of 1327 adults.

### 2.3 Measures

#### 2.3.1 Socio-demographics

Participants were asked to provide information about their age (in years), sex, ethnicity (white/non-white), marital status (married or living with partner; yes/no), social grade assessed with the reduced National Statistics Socio-economic Classification measure [34] and categorised into ABC1/C2DE, education (post-16 [post high school] qualification; yes/no) and England region (North/Central/South).

#### 2.3.2 Health behaviours

Smoking status was assessed by asking participants if they smoked at all these days (including cigarettes hand-rolled ones, pipes or cigars). Those whose responded that they smoked daily or occasionally were classified as smokers. Respondents who did not smoke now but used to smoke daily or occasionally were classified as ex-smokers. Those who said they had tried in the past but had never been a smoker and those who said they had never smoked at all were classified as never smokers.

Physical activity was assessed by asking participants on how many days in the past week they had engaged in a total of 30 min or more of physical activity, which was enough to raise the breathing rate. Responses were classified as meeting guidelines if participants had exercised on 5 or more days [35].

Overweight was determined using self-reported weight and height of participants and defined as a body mass index ≥ 25 kg/m^2^.

Fruit and vegetable consumption was assessed by asking participants how many portions (80 g serving) of fruit and vegetables they ate over the past month using everyday measures of consumption. Respondents consuming at least five portions per day on average were classified as meeting current guidelines [36].

Alcohol consumption was determined by asking respondents on how many days they drank alcohol in a typical week and how many units of alcohol they drank on a typical day. Respondents consuming on average 14 units or less per week were classified as meeting current guidelines [37].

An aggregated behaviour risk score was used, scoring each health behaviour that did not meet guidelines or which indicated greater risk as 1, except for smoking status where current smoking was scored as 2 and past smoking as 1 [38]. This resulted in an overall health behaviour risk score ranging from 0 to 6 with higher scores indicating greater risk.

#### 2.3.3 Cancer beliefs

To assess beliefs about actual and mythical cancer causes, participants were presented with the closed risk factor questions of the CAM [32] and the CAM—MYthical Causes Scale (CAM-MYCS) [33]. These measures have both been validated using UK populations.

The closed risk factor questions of the CAM ask about 11 known cancer risk factors (active smoking; passive smoking; any alcohol consumption; low fruit and vegetable consumption; any red/processed meat consumption; being overweight; sunburnt more than once as a child; being aged 70 years or older; having a relative with cancer; having an infection with HPV; low physical activity). The CAM-MYCS measure asks about 12 factors commonly believed to cause cancer for which there is no scientific evidence (drinking from plastic bottles; eating food containing artificial sweeteners; eating genetically modified food; eating food containing additives; using microwave ovens; using aerosol containers; using mobile phones; using cleaning products; living near power lines; feeling stressed; physical trauma; exposure to electromagnetic frequencies, i.e. non-ionizing radiation of low and high frequencies such as WiFi and Radio/TV frequencies). For both the CAM and CAM-MYCS, participants are asked ‘How much do you agree that each of these can increase a person's chance of developing cancer?’ with response options on a five-point Likert scale (strongly disagree, disagree, unsure, agree, strongly agree).

For the purposes of this analysis, responses were coded as follows. CAM and CAM-MYCS items were dichotomised into ‘correct’ (strongly agree/agree for CAM; strongly disagree/disagree for CAM-MYCS) and ‘incorrect’ (unsure/disagree/strongly disagree for CAM; unsure/agree/strongly agree for CAM-MYCS) responses. This resulted in a total score of 0–11 and 0–12 for the CAM and CAM-MYCS, respectively. The total score was converted to a ‘percentage correct’ (0–100) score, using the percent of maximum possible method to ensure comparability of both scales. The dichotomised ‘correct’ CAM and CAM-MYCS responses were also added together, resulting in a 0–23 CAM total score, converted into ‘percentage correct’ (0–100) score as before. Because data were approximately normally distributed, no further conversions were required.

### 2.4 Analysis

Bivariate associations were assessed with chi-squared test, *t*-test, correlations and analysis of variance as appropriate. General linear models with an identity link were used to determine independent associations of socio-demographic and health behaviour variables with cancer beliefs. For prevalence data, weights were applied to adjust for sampling bias in relation to age, gender, government region, social grade and working status derived from the English 2011 census, Office for National Statistics 2013 mid-year estimates and a random probability survey conducted in 2014 for the National Readership Survey. Family-wise error rate was corrected using the false discovery rate [39], and multiple comparisons were controlled for using the Sidak correction in post hoc analysis. All analyses were carried out in SPSS 24.0.

## 3 Results

Sample characteristics are provided in Table 1, Table 2. On average, participants provided a correct response to a significantly higher proportion of CAM (53%; 95% confidence interval [CI] 51–53) than CAM-MYCS (36%; 95% CI 34–37) items (t[1329] = 15; *P* < 0.01). There was a strong negative correlation between CAM and CAM-MYCS scores (r = –0.43, *P* < 0.01), suggesting that better performance on one measure was associated with worse performance on the other. The combined CAM total score indicated that, on average, fewer than half of items were correctly classified as either cancer causing or not cancer causing (44%; 95% CI 43–45).

**Table 1:** Socio-demographic characteristics of sample and their association with cancer beliefs (N = 1330 adults in England).

Socio-demographic characteristics	Total sample % (N)	CAM	CAM-MYCS	CAM total
		% correct (95% CI)		
**Age**				
Mean (SD)	43.7 (15.3)	–	–	–
≤30	25.7 (352)	52.0 (49.6–54.4)	37.1 (34.0–40.2)	44.2 (42.4–46.0)
31–40	18.4 (244)	52.2 (49.3–55.2)	36.3 (32.7–39.9)	43.9 (41.8–46.0)
41−50	19.7 (263)	52.7 (49.9–55.6)	35.1 (31.6–38.7)	43.5 (41.5–45.6)
51−60	18.1 (241)	54.9 (51.7–58.1)	33.8 (30.1–37.5)	43.9 (41.7–46.1)
≥61	18.1 (240)	49.8 (46.6–52.9)	35.5 (32.0–39.0)	42.3 (40.3–44.3)
**Sex**		a	a	
Male	48.4 (644)	50.7 (48.9–52.5)	38.1 (35.8–40.4)	44.1 (42.8–45.5)
Female	51.6 (687)	53.8 (52.0–55.6)	33.4 (31.3–35.5)	43.2 (42.0–44.4)
**Ethnicity**^b^			a	a
White	84.7 (1122)	52.7 (51.3–54.1)	37.6 (35.9–39.3)	44.8 (43.9–45.8)
Other	15.3 (203)	50.5 (47.2–53.7)	25.3 (21.7–28.9)	37.3 (35.0–39.6)
**Marital status**				
Married/living with partner	63.2 (841)	53.1 (51.4–54.7)	34.6 (32.7–36.5)	43.4 (42.3–44.5)
Single	36.8 (489)	51.0 (49.0–53.1)	37.6 (34.9–40.2)	44.0 (42.5–45.5)
**Social grade**		a		a
ABC1	56.9 (757)	55.7 (54.1–57.3)	36.0 (34.0–38.0)	45.4 (44.3–46.6)
C2DE	43.1 (574)	47.8 (45.8–49.8)	35.2 (32.8–37.6)	41.2 (39.9–42.6)
**Education**^c^		a		a
Post-16 qualification	67.0 (884)	55.4 (53.9–56.9)	35.0 (33.1–36.8)	44.7 (43.6–45.8)
No post-16 qualification	33.0 (435)	46.3 (44.0–48.6)	36.9 (34.1–39.8)	41.4 (39.8–43.0)
**Regions of England**			a	a
North	27.8 (370)	52.7 (50.2–55.1)	40.6 (37.5–43.7)	46.4 (44.8–48.0)
Central	30.1 (400)	51.0 (48.6–53.3)	35.7 (33.0–38.5)	43.0 (41.4–44.7)
South	42.1 (560)	53.0 (51.0–55.0)	32.4 (30.0–34.7)	42.2 (40.8–43.6)

CI, confidence interval; SD, standard deviation; CAM, Cancer Awareness Measure; CAM-MYCS, Cancer Awareness Measure—MYthical Causes Scale; CAM total, aggregated Cancer Awareness Measures.

a Significant differences within category at *P* < 0.05.

b 3 cases missing.

c 11 cases missing.

**Table 2:** Health behaviour characteristics of sample and their association with cancer beliefs (N = 1330 adults in England).

Health behaviour characteristics	Total sample % (N)	CAM	CAM-MYCS	CAM total
		% correct (95% CI)		
**Smoking status**^b^		a	a	
Never smoker	69.7 (921)	54.0 (52.5–55.6)	33.4 (31.6–35.2)	43.3 (42.2–44.3)
Ex-smoker	14.0 (185)	53.9 (50.8–57.1)	39.0 (34.8–43.1)	46.1 (43.8–48.4)
Current smoker	16.3 (216)	43.7 (40.3–47.1)	43.1 (38.8–47.4)	43.4 (40.9–45.8)
**Physical activity**^b^				
≥150 min of exercise per week	31.3 (413)	54.2 (52.0–56.5)	35.5 (32.7–38.2)	44.4 (42.9–46.0)
<150 min of exercise per week	68.3 (909)	51.4 (49.8–53.0)	35.8 (33.9–37.7)	43.2 (42.1–44.3)
**Body mass index**^c^				
<25 kg/m^2^	48.6 (554)	54.0 (52.0–56.0)	36.0 (33.6–38.4)	44.6 (43.2–46.0)
≥25 kg/m^2^	51.4 (586)	51.5 (49.7–53.3)	35.1 (32.7–37.4)	42.9 (41.6–44.2)
**Fruit/vegetable consumption**^d^		a		a
≥5 portions per day	35.9 (475)	58.4 (56.4–60.3)	34.7 (32.3–37.1)	46.0 (44.7–47.4)
<5 portions per day	64.1 (848)	48.9 (47.3–50.6)	36.3 (34.2–38.3)	42.3 (41.2–43.5)
**Alcohol consumption**^e^			a	a
≤14 units per week	86.6 (1128)	52.1 (50.7–53.4)	34.9 (33.2–36.6)	43.1 (42.1–44.0)
>14 units per week	13.4 (174)	53.5 (49.8–57.1)	41.8 (37.5–46.1)	47.4 (44.7–50.0)
**Aggregated behaviour risk score**^f^		a	a	
0–1	22.5 (249)	59.1 (56.3–61.9)	32.8 (29.6–36.1)	45.4 (43.5–47.3)
2–3	59.1 (655)	52.2 (50.5–53.9)	34.8 (32.6–34.0)	43.1 (41.9–44.4)
4–6	18.5 (205)	45.9 (42.5–49.4)	41.7 (37.4–46.0)	43.7 (41.3–46.2)

CI, confidence interval; CAM, Cancer Awareness Measure; CAM-MYCS, Cancer Awareness Measure—MYthical Causes Scale; CAM total, aggregated Cancer Awareness Measure

a Significant differences within category at *P* < 0.05.

b 8 cases missing.

c 190 cases missing.

d 7 cases missing.

e 28 cases missing.

f 220 cases missing.

**Fig. 1:**
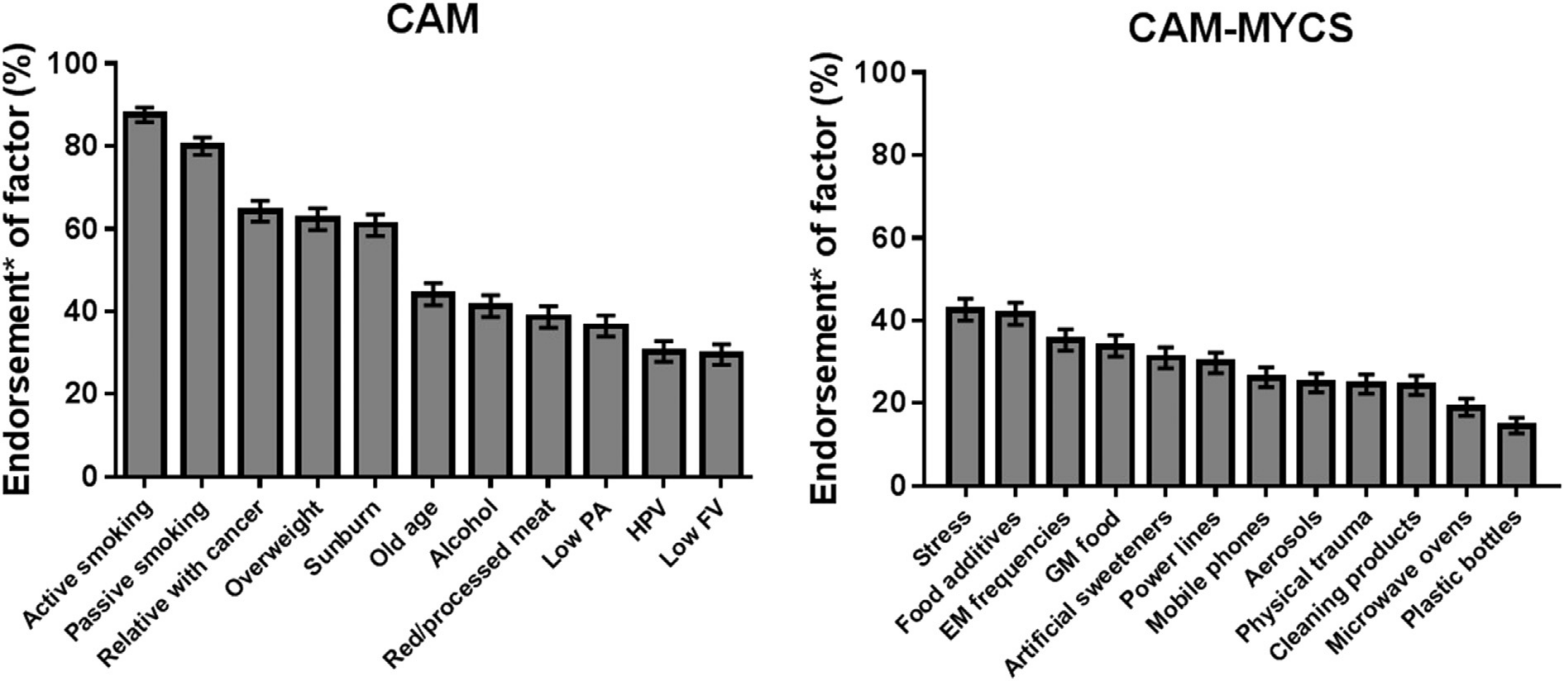
Endorsement of actual and mythical causes of cancer in England. *This is the percentage of participants who chose either ‘agree’ or ‘strongly agree’ for actual (CAM) or mythical (CAM-MYCS) cancer causes. PA, physical activity; HPV, Human Papillomavirus; FV, fruit and vegetable consumption; GM, genetically modified; EM, electromagnetic non-ionizing radiation of low and high frequencies such as WiFi and Radio/TV frequencies; CAM, Cancer Awareness Measure; CAM-MYCS, Cancer Awareness Measure—MYthical Causes Scale.

The most commonly endorsed actual cancer causes (CAM items) were active smoking (88%; 95% CI 86–90) and passive smoking (80%; 95% CI 78–82). By contrast, fewer than a third of participants correctly identified infection with HPV (30%; 95% CI 28–33) or low fruit and vegetable consumption (30%; 95% CI 27–32) as causes of cancer (Fig. 1). The most commonly endorsed mythical cancer causes (CAM-MYCS items) were stress (43%; 95% CI 40–45), food additives (42%; 95% CI 39–44), exposure to non-ionizing electromagnetic frequencies (35%; 95% CI 33–38) and eating genetically modified food (34%; 95% CI 31–36). Fewer than a fifth of participants endorsed using microwave ovens (19%; 95% CI 17–21) or drinking from plastic bottles (15%; 95% CI 13–17) as causing cancer (Fig. 1).

A number of socio-demographic and health behaviour characteristics were associated with CAM, CAM-MYCS and CAM total scores in univariate analyses (Table 1, Table 2). To disentangle the independent associations of these characteristics with cancer beliefs, multivariate analyses were conducted. Better knowledge of actual causes of cancer was associated with white ethnicity, having post-16 qualifications and with greater adherence to health behaviour guidelines/having a lower behaviour risk score (CAM, Table 3). In particular, higher CAM scores were associated with reduced likelihood of current smoking (B = –6.7, 95% CI −11,−2.6; *P* < 0.01) or not eating five portions of fruit and vegetables a day (B = –7.1, 95% CI −9.9,−4.3; *P* < 0.01).

**Table 3:** Independent associations of sample socio-demographic and health behaviour characteristics with cancer beliefs (N = 1082 adults in England).

Sample characteristics	CAM	CAM-MYCS	CAM total
	Adjusted B (95% CI); *P*		
**Age** (years)	0.05 (–0.0, 0.15); 0.25	***–0.18 (***−***0.30,***−***0.07); <0.01***	***−0.07 (***−***0.13,***−***0.01); 0.04***
**Sex**			
Male (ref)	1	1	1
Female	2.3 (−0.5,5.0); 0.11	***−3.7 (***−***7.1,***−***0.32); 0.03***	−0.86 (−2.8,1.1), 0.39
**Ethnicity**			
White (ref)	1	1	1
Other	***−5.4 (***−***9.3,***−***1.5); 0.01***	***−11.7 (***−***16.1,***−***6.9); <0.01***	***−8.6 (***−***11.5,***−***5.6); <0.01***
**Marital status**			
Married/living with partner (ref)	1	1	1
Single	1.7 (−1.2,4.6); 0.25	0.96 (−2.7,4.6); 0.61	1.3 (−0.73,3.4); 0.21
**Social grade**			
ABC1 (ref)	1	1	1
C2DE	−2.7 (−5.7,0.2); 0.07	−1.8 (−5.4,1.9); 0.35	***−2.2 (***−***4.3,***−***0.11); 0.04***
**Education**			
Post-16 qualification (ref)	1	1	1
No post-16 qualification	***−5.5 (***−***8.7,***−***2.3); <0.01***	0.36 (−3.8,4.5); 0.86	***−2.5 (***−***4.7,***−***0.15); 0.04***
**Region of England**			
North (ref)	1	1	1
Central	−2.1 (−5.8,1.7); 0.28	−2.6 (−7.2,1.9); 0.26	−2.4 (−4.9,0.17); 0.07
South	0.54 (−2.9,4.0); 0.76	***−5.1 (***−***9.4,***−***0.70); 0.02***	−2.4 (−4.8,0.00); 0.05
**Aggregated behaviour risk score**			
0–1 (ref)	1	1	1
2–3	***−5.7 (***−***9.0,***−***2.3); <0.01***	1.6 (−2.5,5.7); 0.44	−1.9 (−4.2,0.52); 0.13
4–6	***−11.7 (***−***16.2,***−***7.1); <0.01***	***6.8 (1.2,12.5); 0.02***	−2.0 (−5.3,1.2); 0.22

CI, confidence interval; CAM, Cancer Awareness Measure; CAM-MYCS, Cancer Awareness Measure—MYthical Causes Scale; CAM total, aggregated Cancer Awareness Measures; ref, reference group.

Significant associations are in bold italics.

By contrast, better knowledge of mythical causes of cancer was independently associated with being younger, male, white and from the North (versus South) of England (CAM-MYCS, Table 3). In addition, higher CAM-MYCS scores were related to a higher aggregated behaviour risk score; specifically, current (B = 8.0, 95% CI 2.8,13; *P* < 0.01) or past smoking (B = 5.0, 95% CI 0.1,10; *P* < 0.05) was associated with higher scores on the CAM-MYCS.

For the overall combined CAM total, greater knowledge of both actual and mythical causes of cancer was independently associated with younger age, being white, from a higher socio-economic group and having post-16 qualifications (CAM total, Table 3). Respondents who performed worse on the CAM total were less adherent to fruit and vegetable consumption guidelines (B = –3.1, 95% CI −5.1,−1.1; *P* < 0.01), but it was not associated with any other health behaviour.

## 4 Discussion

This is the first study to provide population data on beliefs about a range of actual and mythical causes of cancer. In contrast with previous work [16], participants showed relatively poor awareness of factors that are not causally linked to cancer, with only a third of mythical cancer causes identified as such. Stress, food additives, genetically modified foods and exposure to non-ionizing electromagnetic frequencies were actively endorsed as causing cancer by more than a third of participants. Endorsement of mythical causes of cancer appears to have increased over the last decade. This may be a reflection of changes in the way people access news [40] or could result from methodological differences between our study and previous work (e.g. 16).

Knowledge of risk factors causally linked to cancer was higher than knowledge of factors not causally linked to cancer, but still disappointingly low. Low fruit and vegetable consumption was the least recognised cancer risk factor, with less than one third of participants reporting it. Obesity was also poorly recognised, which is concerning considering it is the second leading preventable cause of cancer [1]. Similar observations have been made in a number of European countries, highlighting an area of concern throughout the region [17], [18], [21], [22], [23], [41]. Raising awareness of the role of weight in cancer development is likely to be an essential first step in the behaviour change sequence.

These estimates can be used to benchmark public understanding of cancer risk factors. Future comparisons can be made with these data to monitor improvements and reductions in cancer awareness. Historically, such comparisons have relied on unvalidated survey tools that do not include mythical risk factors. The CAM-MYCS allows for reliable measurement of beliefs about mythical cancer causes which will be useful in the evaluation of future public health programmes. The scale could also be used to investigate whether cancer survivors attribute their disease to known or mythical factors. Such data could be used to reassure patients, particularly those who experience stigma or feel a sense of blame regarding their disease [42], [43]. Indeed, mythical beliefs might be associated with raised anxiety, especially where the source of the risk is outside the individual's control [44], [45]. By improving the accuracy of people's causal beliefs, we might be able to reduce cancer fear or worry and make people feel more empowered about their ability to reduce their risk.

In line with previous research, participants who were white and had spent longer in education were more likely to identify actual cancer causes [16], [18], [25]. Younger age and white ethnicity were associated with better identification of mythical risk factors. When combining responses to assess beliefs about both actual and mythical cancer causes, lower age, white ethnicity, higher social grade and longer time spent in education were all independently associated with better awareness of factors that do and do not cause cancer. These patterns broadly reflect previous work [16], [17], [18], indicating that traditionally underserved populations are at risk of having a poorer understanding of cancer risk factors. Monitoring awareness of known and mythical risk factors is important to ensure socio-economic inequalities in cancer knowledge are not widening. These findings also highlight that information should be tailored to the needs of those who lack awareness; for instance, by using graphical, simple ways to communicate risk to people with limited formal schooling and literacy.

Lower awareness of known risk factors was associated with a greater likelihood of smoking and not adhering to the fruit and vegetable consumption guidelines. By contrast, better awareness of mythical factors was associated with a greater likelihood of smoking and having a higher aggregated behaviour risk score. The combined measure of awareness of actual and mythical cancer risk factors indicated that a better understanding of cancer aetiology was associated with adequate fruit and vegetable consumption. These complex set of associations require replicating in additional samples internationally before definitive recommendations can be made regarding the link between knowledge of cancer risk factors and preventive behaviours.

Incorrectly endorsing mythical causes of cancer was not associated with engaging in riskier health behaviours in our study. This is reassuring insofar as it would suggest that simply holding incorrect beliefs about mythical cancer causes does not necessarily result in poorer lifestyle choices. However, the fact that a third of participants believe that factors such as stress, genetically modified food, food additives and non-ionizing electromagnetic frequencies cause cancer highlights the need to continue monitoring risk perceptions [46]. Investigating associations between the CAM, CAM-MYCS and other cancer control behaviours, such as cancer screening participation and early presentation with symptoms, would be a useful next step for future research.

The finding that those who were better at identifying actual causes of cancer were also worse at identifying mythical causes of cancer was unexpected. One possible explanation may be that some people are generally more likely to make causal attributions to cancer, whether or not these are actually true. This may result in more health-protective behaviours to avoid disease [26], as reflected by lower smoking rates and greater consumption of fruits and vegetables among respondents who endorsed both actual and mythical cancer causes. By the same token, if people engage in unhealthy behaviours, they may be more likely to downplay any potential causal associations (whether or not they are accurate) with cancer. This may explain why those who correctly rejected mythical causes of cancer (but also incorrectly rejected actual causes of cancer) were more likely to be smokers. Such risk denial by smokers has also been observed elsewhere [47].

This study has limitations. These data were cross sectional and therefore we cannot make causal claims about the associations between cancer beliefs and socio-demographic or behavioural correlates. Further research would benefit from longitudinal and experimental work to determine the direction of this association and whether or not improving awareness of actual or mythical causes of cancer, or both, influences health behaviour choices. Moreover, both measures of beliefs about actual and mythical causes of cancer used in our study may require validation in other countries across Europe to ensure universality of the identified factors. This is particularly important, given the observed associations with ethnicity. Relatedly, we did not measure religiosity or fatalism as explanatory correlates which are likely to influence people's views on what does or does not cause cancer. In addition, the items included were risk factors attributed to cancer generally, rather than being site specific. As was performed with the CAM, site-specific versions of the CAM-MYCS can be developed in the future. Finally, the measures of health behaviours used in this study were self-reported and may, therefore, be biased assessments of adherence to health behaviour guidelines.

## 5 Conclusions

Knowledge of actual causes of cancer is greater in the general population than that of mythical causes. However, awareness was generally low for both types of factor, which likely has implications for efforts to promote cancer prevention in the general population. The pattern of associations between socio-demographic groups and awareness of actual and mythical risk factors was inconsistent but should continue to be monitored to ensure inequalities in cancer knowledge are not widening. Engagement in health-protective behaviours is associated with accurate beliefs about actual cancer causes but shows no association with endorsement of mythical causes.

## Conflict of interest statement

L.S. has received honoraria for talks, an unrestricted research grant and travel expenses to attend meetings and workshops by pharmaceutical companies that make smoking cessation products (Pfizer, Johnson & Johnson), has acted as a paid reviewer for grant awarding bodies and as a paid consultant for healthcare companies. Other research has been funded by the government, a community-interested company (National Centre for Smoking Cessation and Training) and charitable sources. The other authors declare that they have no conflict of interest to disclose.

## Author contribution statement

S.G.S. and L.S. conceived the original idea for this study. S.G.S and L.S. obtained funding. J.A.M., S.G.S. and L.S. managed the day-to-day running of the study. L.S. undertook the data analyses and wrote the initial draft with further input from all authors. L.S. is the guarantor for this article. L.S., J.A.M., J.W. and S.G.S. reviewed and approved the final version. All researchers listed as authors are independent from the funders and all final decisions about the research were taken without constraint by the investigators. L.S. had full access to all the data in the study and had final responsibility for the decision to submit for publication.

## Funding

This work was supported by a Cancer Research UK/Bupa Foundation Innovation Award (C42785/A20811). S.G.S. and J.W. are supported by Cancer Research UK fellowships (C42785/A17965 and C7492/A17219, respectively). L.S. is a member of the UK Centre for Tobacco and Alcohol Studies (UKCTAS), funded under the auspices of the aforementioned UK Clinical Research Collaboration (MR/K023195/1). The funders had no role in the collection, analysis or interpretation of data; in the writing of the report or in the decision to submit the article for publication.
